# Isodeoxyelephantopin induces protective autophagy in lung cancer cells via Nrf2-p62-keap1 feedback loop

**DOI:** 10.1038/cddis.2017.265

**Published:** 2017-06-15

**Authors:** Yang Wang, Jing Zhang, Zhi-Hao Huang, Xiao-Hui Huang, Wei-Bin Zheng, Xing-Feng Yin, Yao-Lan Li, Bin Li, Qing-Yu He

**Affiliations:** 1 Key Laboratory of Functional Protein Research of Guangdong Higher Education Institutes, Institute of Life and Health Engineering, College of Life Science and Technology, Jinan University, Guangzhou, China; 2 Institute of Traditional Chinese Medicine and Natural Products, College of Pharmacy, Jinan University, Guangzhou, China

## Abstract

Isodeoxyelephantopin (ESI), isolated from *Elephantopus scaber* L. has been reported to exert anticancer effects. In this study, we aimed to investigate whether and how cancer cells exert protective responses against ESI treatment. Confocal fluorescence microscopy showed that ESI significantly induced autophagy flux in the lung cancer cells expressing mCherry-EGFP-LC3 reporter. Treatment of the cells with ESI increased the expression levels of the autophagy markers including LC3-II, ATG3 and Beclin1 in a dose-dependent manner. Pretreatment with autophagy inhibitor 3-methyladenine (3-MA) not only attenuated the effects of ESI on autophagy, but also enhanced the effects of ESI on cell viability and apoptosis. Mechanistically, the SILAC quantitative proteomics coupled with bioinformatics analysis revealed that the ESI-regulated proteins were mainly involved in Nrf2-mediated oxidative stress response. We found that ESI induced the nuclear translocation of Nrf2 for activating the downstream target genes including HO-1 and p62 (SQSTM1). More importantly, ESI-induced p62 could competitively bind with Keap1, and releases Nrf2 to activate downstream target gene p62 as a positive feedback loop, therefore promoting autophagy. Furthermore, knockdown of Nrf2 or p62 could abrogate the ESI-induced autophagy and significantly enhanced the anticancer effect of ESI. Taken together, we demonstrated that ESI can sustain cell survival by activating protective autophagy through Nrf2-p62-keap1 feedback loop, whereas targeting this regulatory axis combined with ESI treatment may be a promising strategy for anticancer therapy.

Lung cancer is the leading cause of cancer-related deaths around the world and non-small cell lung cancer (NSCLC) was especially considered as the most common form, accounting for approximately 85% of all lung cancer cases.^1^ The 5-year survival rate of NSCLC remains as low as about 15% because of fast growth, early metastases and the resistance to chemotherapy and radiotherapy.^2^ In the past decades, chemotherapeutic agents such as cisplatin and docetaxel were widely applied in clinic. However, NSCLC is a malignant disease with activation of multiple major signaling pathways, which results in cancer cell survival and chemoresistance.^3^ Development of novel therapeutic agents is urgently needed to treat this lethal disease, and multidrug combination strategy is regarded as a promising way in cancer therapy.

Natural products have been used for the prevention and cure of diseases for centuries, particularly in cancer therapy. Increasing natural active ingredients derived from medicinal herbs were successfully applied to cancer therapy in clinic.^4, 5, 6^ *Elephantopus scaber* L. is a popular medicinal herb and its antiviral and hepatoprotective effects have been documented.^7, 8^ In China, it has been widely used to prevent and treat respiratory disease, especially lung cancer ^9^ and nasopharyngeal carcinoma.^10, 11^ Isodeoxyelephantopin (ESI) and deoxyelephantopin (ESD), the two sesquiterpene lactones isolated from *Elephantopus scaber* L,^12, 13, 14, 15^ have been reported to exert antitumor effects in several malignant carcinomas.^16, 17^ Fully understanding the action mechanisms of ESI, in particular, whether there is protective response against ESI treatment in cancer cells is urgently needed for minimization of the dosage in preclinical experiment and development of combined therapeutic strategies.

Autophagy, interpreted as cell 'self-eating', is a highly evolutionarily conserved catabolic process in eukaryotes, having vital roles in regulation of protein homeostasis, and is essential for survival when cells face metabolic stress.^18^ The whole autophagic process was regulated by series of signaling pathways including the autophagy-related gene (ATG) family,^19^ adenosine monophosphate-activated protein kinase^20^ and the phosphatidylinositol 3-kinase/AKT/mammalian target of rapamycin pathway.^21^ Increasing evidences demonstrated that autophagy induced by chemotherapy or radiotherapy may prevent cancer cells from apoptosis, leading to unfavorable conditions in anticancer therapy.^22, 23^

Apart from autophagy, another cellular protective signaling is nuclear factor erytheroid-derived-2-like 2 (Nrf2), which can confer adaptive protection against oxidative and proteotoxic stress in cells.^24, 25, 26^ In the resting status of cells, Nrf2 is carried to proteasome by keap1 for degradation;^27, 28^ upon oxidative stress, the Nrf2 released from Nrf2-keap1 complex translocates to nucleus and then activates the transcription of downstream target genes.^28^ Emerging evidences showed that Nrf2 can promote the resistance of cancer cells to chemotherapeutic drugs,^29^ whereas knockdown of Nrf2 signaling by small interfering RNA (siRNA) or small molecules, such as brusatol,^30^ rendered cancer cells more susceptible to chemotherapeutic agents. It has been reported that Nrf2 signaling is alternatively activated to promote cell survival once the autophagic flux is dysregulated,^23^ but the synergistic effect of the two biological progresses remains unknown.

Previous study from our laboratory has demonstrated that ESI can induce cell apoptosis through ROS-dependent DNA damage and antitumor inflammation factor pathway.^10^ In this study, the unexpected finding that ESI could induce protective autophagy through Nrf2-p62-keap1 feedback loop to sustain lung cancer cell survival, suggests that blockade of this feedback loop in combination with ESI is a promising strategy for lung cancer therapy.

## Results

### ESI suppressed the growth of lung cancer cells

**Figure 1:**
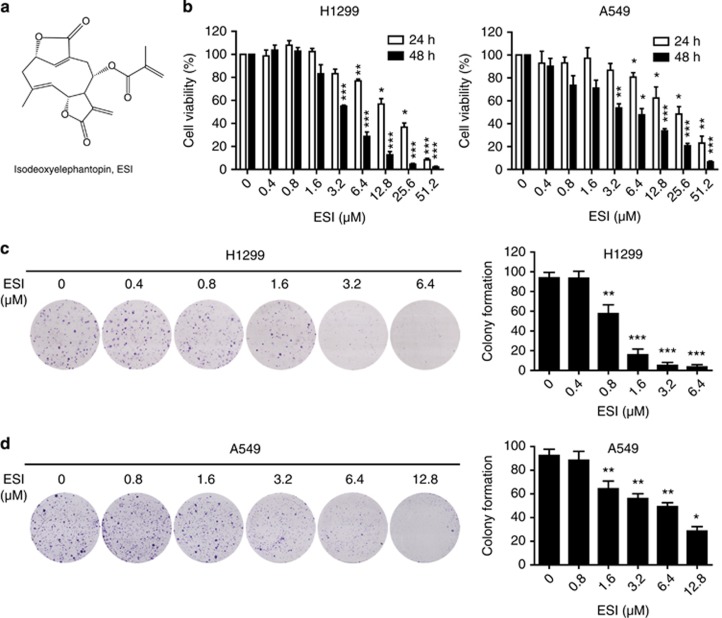
ESI inhibits the growth of lung cancer cells H1299 and A549. (**a**) The molecular structure of ESI. (**b**) Cells were incubated with various concentrations (up to 51.2 *μ*M) of ESI for 24 and 48 h and their viability was then determined by WST-1 assay. (**c** and **d**) ESI inhibited the colony formation ability of H1299 and A549 cells. All data were representative of three independent experiments. Bars, S.E.M.; *P<0.05, **P<0.01, ***P<0.001

The chemical structure of ESI is shown in Figure 1a. Before investigation of the pharmacological potential of ESI, we determined the cytotoxicity of ESI by treating lung cancer cells, H1299 and A549, with ESI at various concentrations ranging from 0 to 51.2 *μ*M for 24 and 48 h. As shown in Figure 1b, ESI treatment resulted in significantly decreased cell viability in H1299 and A549 cells in dose- and time-dependent manners. Intriguingly, ESI was found to have markedly less cytotoxicity toward non-cancer lung epithelial cells HBE (Supplementary Figure S1A). To further determine the effects of ESI on the growth of lung cancer cells, we conducted colony formation assay in both H1299 and A549 cell lines. As shown in Figures 1c and d, H1299 and A549 cells were treated with different concentrations of ESI, and their colony numbers were quantified and statistically analyzed. Consistently, ESI significantly inhibited colony formation ability of H1299 and A549 cells but not in HBE (Supplementary Figure S1B), indicating that ESI has potently inhibitory effect on lung cancer cell growth.

### ESI induces autophagy in lung cancer cells

**Figure 2:**
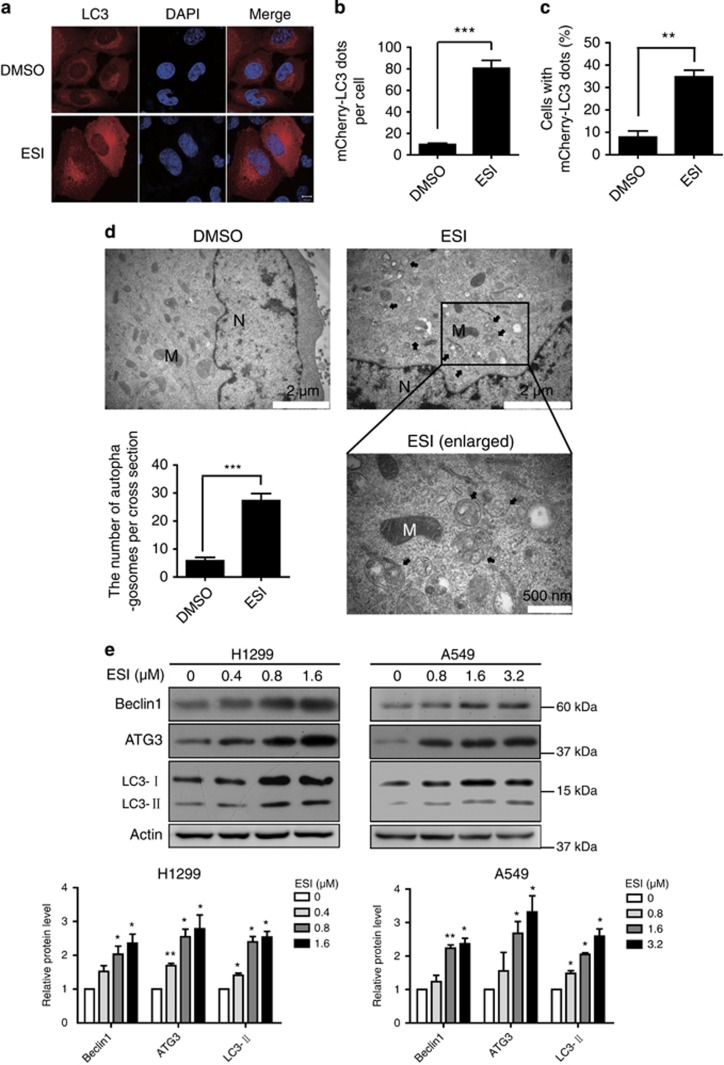
ESI induces the formation of autophagic vacuoles in H1299 and A549 cells. (**a**) H1299 cells overexpressing mCherry-LC3 were treated with DMSO or ESI (1.6 *μ*M) for 24 h, and the mCherry-LC3 punctate dots in cells were examined. Positive signals were defined if the cells have five or more mCherry-LC3 dots in the cytoplasm. (**b** and **c**) The number of mCherry-LC3 dots per cell (**b**) and the percentage of the cells with mCherry-LC3 dots (**c**) were counted under florescence microscope. (**d**) Ultrastructural features of the H1299 cells treated with ESI (1.6 *μ*M) for 24 h were analyzed by electron microscopy. The typical images of nucleus (N), mitochondria (M) and autophagosomes (arrows) were shown at higher magnification. The number of autophagosomes in H1299 cells was presented. Twenty cross-sections were counted in each experiment. (**e**) H1299 and A549 cells were treated with DMSO or indicated concentrations of ESI for 24 h, and then compared for expression levels of Beclin1, ATG3 and LC3 by western blot. Actin was included as loading control. All data were representative of three independent experiments. Bars, S.E.M.; *P<0.05, **P<0.01, ***P<0.001

As the anticancer effect of 24-h ESI treatment at lower concentrations (0.4, 0.8, 1.6 *μ*M for H1299 cells and 0.8, 1.6, 3.2 *μ*M for A549 cells) is very slight, we aimed to reveal the molecular mechanisms and uncover whether there is protective cellular response in lung cancer cells, therefore, we chose these concentrations and time points for further investigation. The recruitment of LC3-II to autophagosomes in response to ESI treatment was determined by examining the appearance of a punctate mCherry-LC3 signal in H1299 cells. As shown in Figure 2a, confocal fluorescence microscopy analysis demonstrated that punctate mCherry-LC3 staining was observed in the cytoplasm of H1299 cells in response to 1.6 *μ*M of ESI treatment for 24 h, whereas only diffuse LC3-associated red fluorescence could be seen in the DMSO-treated control cells. These mCherry-LC3 dots were quantified and statistically analyzed with significance (Figures 2b and c). Furthermore, the ultrastructure of H1299 cells treated with 1.6 *μ*M ESI or DMSO for 24 h was analyzed by transmission electron microscopy. Numerous membrane-bound vacuoles characteristic of autophagosomes were observed in the cytoplasm of ESI-treated cells, but rarely in the cells treated with DMSO (Figure 2d). In addition, immunoblotting analysis of autophagy markers was performed to determine the cellular autophagy induced by ESI. As shown in Figure 2e, the protein levels of ATG3, LC3-ll and Beclin1 increased in a dose-dependent manner in the H1299 and A549 cells treated with low concentrations of ESI.

**Figure 3:**
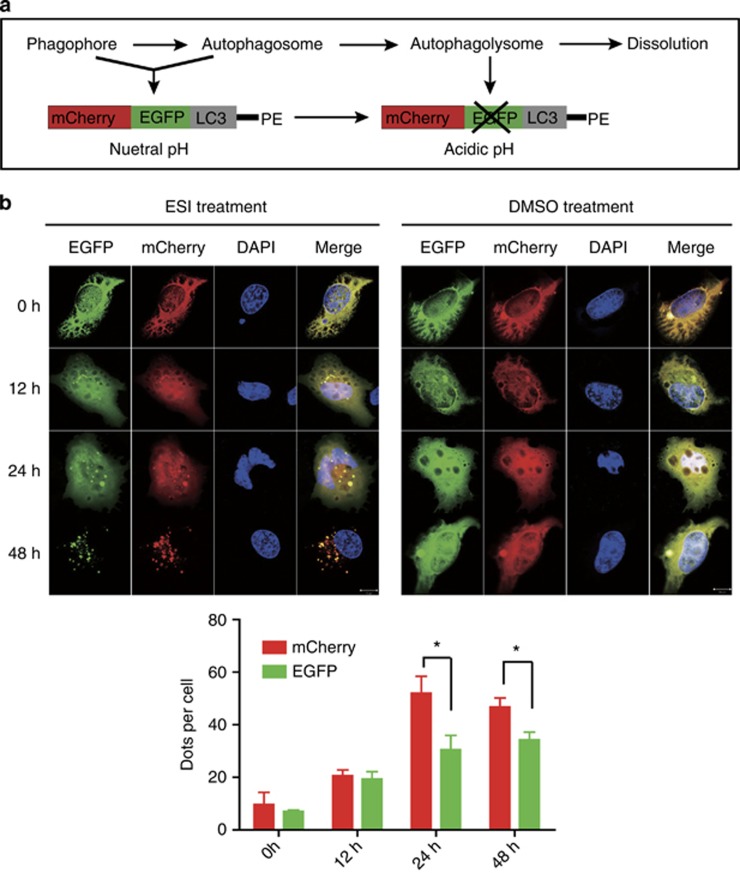
ESI induces the appearance of autophagy flux in lung cancer cells. (**a**) Schematic representation of mCherry-EGFP-LC3 constructs. (**b**) H1299 cells overexpressing mCherry-EGFP-LC3 were treated with 1.6 *μ*M ESI (left) or DMSO (right) for 0, 12, 24, 48 h, respectively, and then subjected to confocal microscopy. Scale bar: 10 *μ*m. The average numbers of green and red LC3 dots per cell in each condition were quantified, and >30 cells were counted in each condition. All data were representative of three independent experiments. Bars, S.E.M.; *P<0.05

To further analyze how ESI affected the stepwise progression of autophagy, we constructed a mCherry-EGFP-LC3 reporter (Figure 3a) to observe the autophagy flux progress. The GFP signal in the mCherry-EGFP-LC3 fusion protein is quenched under acidic pH in autophagolysosomes, which makes it easy to distinguish between autophagosomes and autophagolysosomes.^31, 32^ As shown in Figure 3b, 6–10 yellow spots were observed in the untreated H1299 cells. However, after 12 h of ESI stimuli, red and yellow speckles were accumulated in the cells as compared with control. Furthermore, red spots were predominantly observed rather than green upon 24 h of ESI treatment, suggesting that both autophagosomes and autophagolysosomes were accumulated and unimpaired after ESI treatment. The confocal fluorescent signals were quantified and statistically analyzed as bar plots in Figure 3b. Collectively, these results showed that ESI triggered autophagy in lung cancer cells.

### Blockade of autophagy enhances ESI-induced apoptosis and growth inhibition

**Figure 4:**
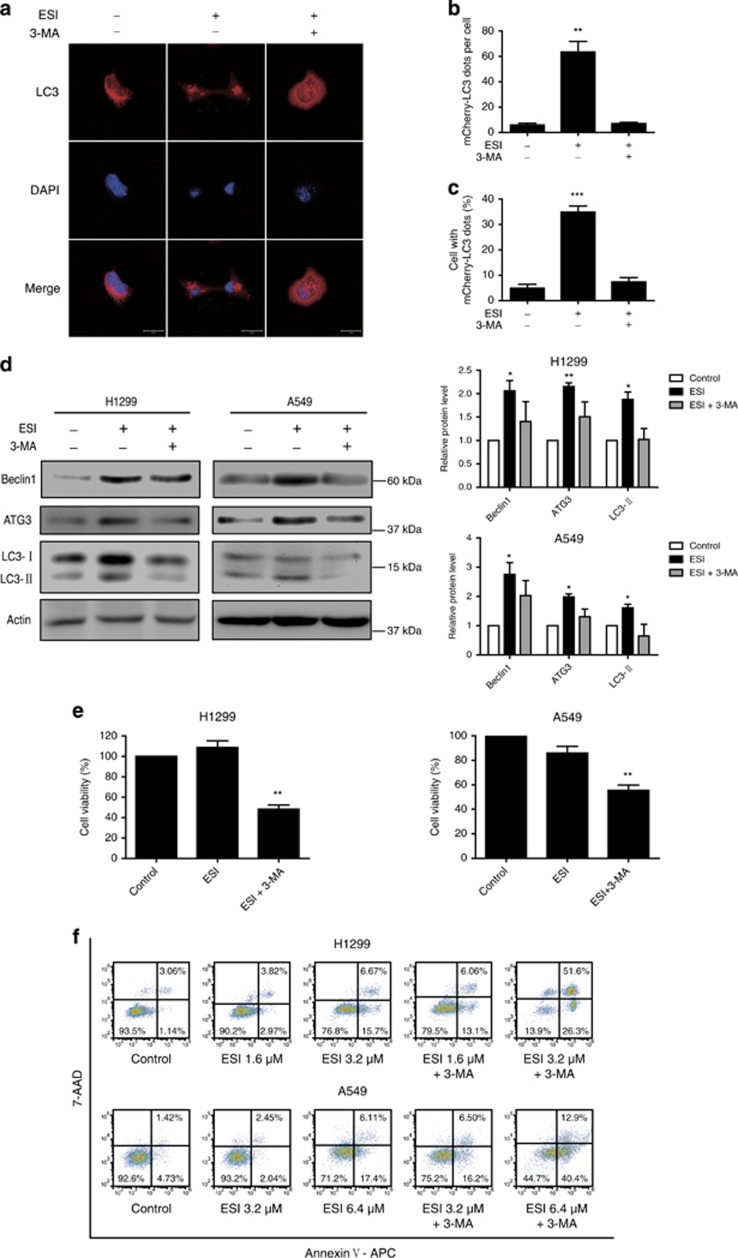
Blockade of autophagy enhances the anticancer effect of ESI in lung cancer cells. (**a**) H1299 cells overexpressing mCherry-LC3 were treated with 1.6 *μ*M ESI in presence or absence of 2 mM 3-MA for 24 h, and the cells with mCherry-LC3 punctate dots were examined. Positive signals were defined if the cell have five or more mCherry-LC3 dots in the cytoplasm. (**b**) The number of mCherry-LC3 dots per cell and (**c**) the percentage of the cells with mCherry-LC3 dots were counted under florescence microscope. (**d** and **e**) Both H1299 and A549 cells were pretreated with 2 mM 3-MA, an inhibitor of autophagy, for 1 h and then exposed to ESI (1.6 *μ*M for H1299, 3.2 *μ*M for A549) for another 24 h. (**d**) Western blot analysis of Beclin1, ATG3 and LC3 expression levels in the H1299 and A549 cells. Actin was used as loading control. (**e**) The cell viability was determined by WST-1 assay. (**f**) H1299 and A549 cells were treated with ESI alone or together with 2 mM 3-MA for 24 h, and then apoptotic cells were detected with the Annexin V-APC/7-AAD kit and analyzed by flow cytometry. All data were representative of three independent experiments. Bars, S.E.M.; *P<0.05, **P<0.01, ***P<0.001

It is widely accepted that autophagy is activated for survival when cell faces stress.^33, 34^ Here, we used 3-methyladenine (3-MA), an inhibitor of autophagy, to evaluate the role of ESI-induced autophagy in lung cancer cells. The confocal analysis showed that exposure of H1299 cells to ESI resulted in cellular punctate mCherry-LC3 accumulation, and this effect was significantly inhibited by 3-MA (Figures 4a–c). In addition, western blot data showed that 3-MA attenuated the effects of ESI on expression levels of ATG3, Beclin1 and LC3 (Figure 4d). Next, we tested whether the pro-survival effect of ESI can be abolished by inhibition of autophagy. As shown in Figure 4e, H1299 and A549 cells were treated with low concentrations of ESI (1.6 *μ*M for H1299 and 3.2 *μ*M for A549) with or without the addition of 3-MA (2 mM) for 24 h, and we found that 3-MA significantly enhanced the anticancer effect of ESI. Moreover, Annexin V/7-AAD assays revealed that treatment of H1299 and A549 cells with both ESI and 3-MA resulted in significantly greater apoptosis than the cells treated with ESI alone (Figure 4f). These data showed that repression of protective autophagy could enhance the effects of ESI in inducing apoptosis and thus inhibit the growth of lung cancer cells.

### Proteomics identifies the activation of Nrf2 signaling induced by ESI

**Figure 5:**
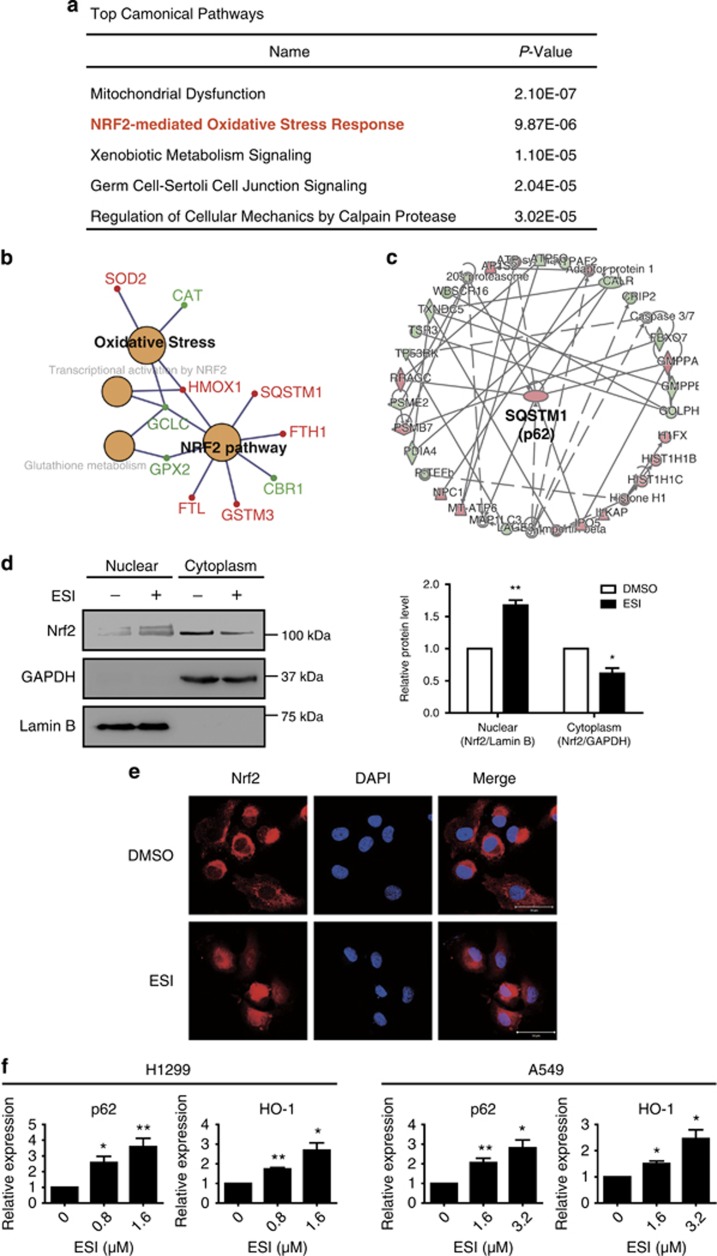
ESI induces the activation of Nrf2 in lung cancer cells. (**a**) The top five canonical pathways involved in the ESI-regulated proteins were identified by quantitative proteomics and IPA analysis. (**b**) Regulatory networks of enriched biological processes of ESI-regulated proteins were analyzed by ClueGO+CluePedia. (**c**) ESI-regulated functional signaling networks were closely associated with SQSTM1 (p62) pathway. (**d**) H1299 cells were treated with ESI (1.6 *μ*M) for 24 h, and then the nuclear and cytoplasmic fractions were isolated for western blot. Lamin B and GAPDH were used as markers for nucleus and cytoplasm, respectively. Note that ESI promoted the nuclear translocation of Nrf2. (**e**) H1299 cells were treated with ESI (1.6 *μ*M) for 24 h and confocal microscopy confirmed the nuclear translocation of Nrf2. Red, Nrf2; Blue, DAPI. Scale bar: 10 *μ*m. (**f**) ESI stimulated the expression levels of p62 and HO-1 at mRNA level, as determined by qRT-PCR. All data were representative of three independent experiments. Bars, S.E.M.; *P<0.05, **P<0.01

The core role of autophagy in promoting cell survival is widely recognized.^35^ Here, to investigate the molecular mechanisms how ESI-induced protective autophagy, we performed SILAC quantitative proteomics to explore the proteins regulated by ESI. Treatment with 3.2 *μ*M ESI for 48 h, but not 24 h, significantly suppressed cell viability in A549 cells (Figure 1). Therefore, to uncover the protective responses of cancer cells to ESI treatment by SILAC quantitative proteomics, we chose a concentration of 3.2 *μ*M and a time point of 24 h for A549 cells so that the treatment could not significantly inhibit cell viability. We believe this setting may avoid the interference of other pathways involved in cell apoptosis or death and help us identify the proteins directly regulated by ESI. A total of 378 proteins were identified to be significantly regulated by ESI (fold change ≥1.3), including 149 upregulations and 229 downregulations (Supplementary Table S1). Ingenuity pathway analysis (IPA) was used to characterize the canonical pathways that the 378 differential proteins participated in. As shown in Figure 5a, Nrf2 signaling, which has an important role in promoting survival of cancer cells, was significantly regulated by ESI treatment. Gene ontology (GO) annotation also suggested that Nrf2 pathway, oxidative stress and glutathione metabolism were linked to ESI treatment (Figure 5b). In addition, a cluster of the ESI-regulated proteins constructed a signaling network that strongly pointed to a hub protein, p62 (Figure 5c), a target gene of Nrf2.^36^ Activation of Nrf2 signaling, indicated by the nuclear translocation of Nrf2, have a critical role in important biological progresses including autophagy, proliferation and metastasis. In this connection, we found that Nrf2 expression was dramatically increased in the nucleus when H1299 cells were exposed to ESI (1.6 *μ*M) (Figure 5d). The ESI-induced nuclear translocation of Nrf2 was then confirmed by confocal microscope assay (Figure 5e). We further showed that ESI significantly increased the expression levels of HO-1 and p62 (Figure 5f), the downstream targets of Nrf2. These experimental results showed that ESI activated Nrf2 signaling pathway in lung cancer cells.

### Nrf2 is required for ESI-induced autophagy

**Figure 6:**
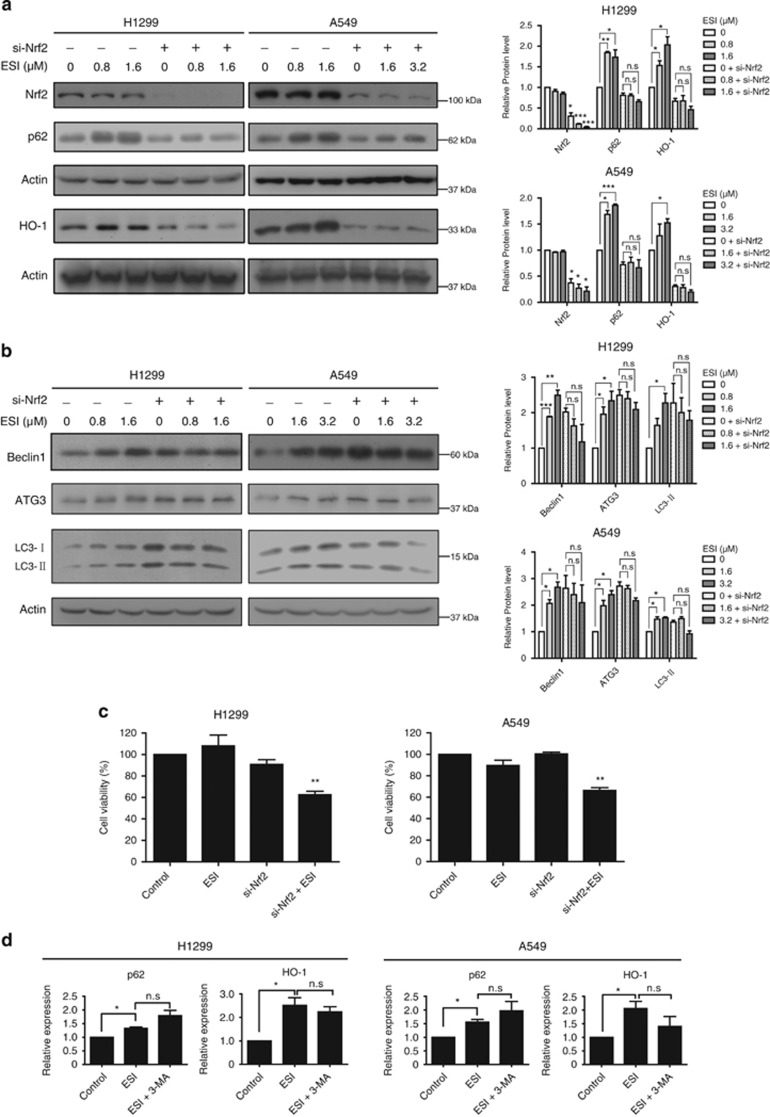
The Nrf2 signaling is required for effect of ESI in inducing autophagy. (**a**–**c**) H1299 and A549 cells transfected with anti-Nrf2 siRNA (50 nM) or control siRNA were treated with ESI, western blot assays were used to determine the expression levels of p62 and HO-1 (**a**) and the autophagy markers including Beclin1, ATG3 and LC3 (**b**), and the cell viability was measured by WST-1 assay (**c**). Note that ESI-induced protective autophagy and cell viability can be attenuated by knockdown of Nrf2. (**d**) Both H1299 and A549 cells were pretreated with 3-MA (2 mM) and then exposed to ESI (1.6 *μ*M for H1299, 3.2 *μ*M for A549) for another 24 h, the mRNA levels of p62 and HO-1 were determined by qRT-PCR. All data were representative of three independent experiments. Bars, S.E.M.; *P<0.05, **P<0.01, ***P<0.001; Nonsignificant is referred as n.s

Previous studies reported that deficiency in autophagy led to prolonged Nrf2 activation,^23^ however, our present data demonstrated that both autophagy and Nrf2 signaling were activated by ESI treatment. We thus determined whether a non-canonic signaling network, which includes both autophagy and Nrf2 pathways, is induced by ESI. First, lung cancer cells were treated with ESI with or without transfection of the siRNA against Nrf2, and the protein levels of p62 and HO-1, two target genes of Nrf2, were determined by western blot. The results showed that the increased expression levels of p62 and HO-1 induced by ESI were significantly attenuated in Nrf2-knockdown cells (Figure 6a). Moreover, as shown in Figure 6b, we found that ESI increased expression levels of several autophagic markers including ATG3, Beclin1 and LC3-ll in a dose-dependent manner in both H1299 and A549 cells, whereas the effects were abolished by the pretreatment with Nrf2 siRNA. In addition, knockdown of Nrf2 enhanced the anticancer effects of ESI in both cell lines (Figure 6c). On the other hand, pretreatment with 3-MA did not attenuate the ESI-induced Nrf2 activation, as indicated by the mRNA expression levels of p62 and HO-1, the two target genes of Nrf2 signaling (Figure 6d). These data suggest that Nrf2 activation serves as the upstream regulator and is required for ESI-induced autophagy.

### Nrf2-p62-keap1 feedback loop is involved in ESI-induced protective autophagy

**Figure 7:**
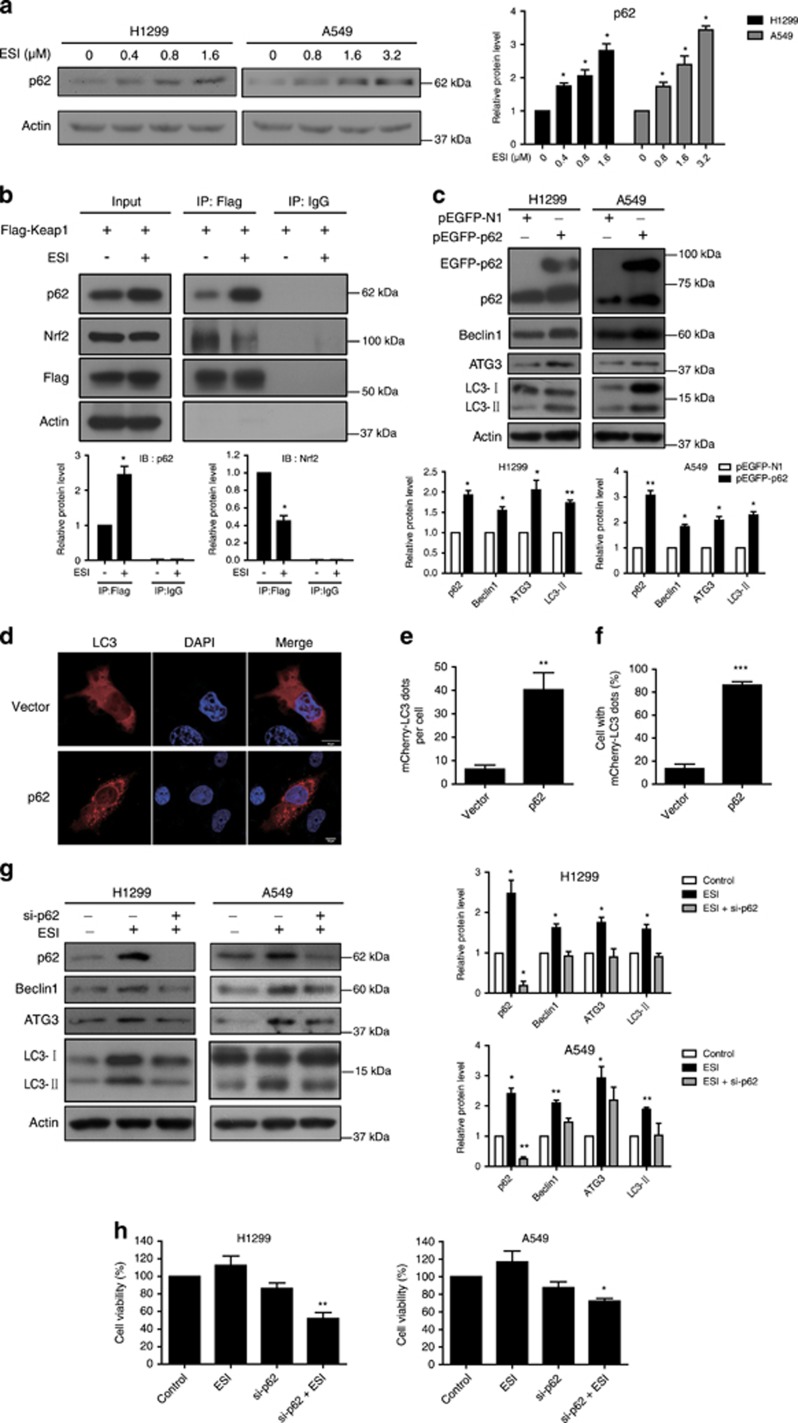
p62 is required for ESI-induced autophagy. (**a**) H1299 and A549 cells were treated with the indicated concentration of ESI for 24 h, and the p62 protein level was determined by western blot. (**b**) H1299 cells transfected with Flag-Keap1 plasmid were treated with ESI (1.6 *μ*M) for 24 h. Immunoprecipitation was performed using an anti-Flag antibody or IgG as control, and immunublotting was carried out on the total cell lysates or immunoprecipitates using the indicated antibodies. (**c**) H1299 and A549 cells were transfected with pEGFP-p62 plasmid and pEGFP-N1 (vector control), and the autophagy markers including Beclin1, ATG3 and LC3 were determined by western blot. (**d**-**f**) H1299 cells overexpressing mCherry-LC3 were transfected with pEGFP-p62 plasmid and pEGFP-N1 (vector control) for 24 h, and the mCherry-LC3 punctate dots in cells were examined. Positive signals were defined if the cells have five or more mCherry-LC3 dots in the cytoplasm. (**e** and **f**) The number of mCherry-LC3 dots per cell (**e**) and the percentage of the cells with mCherry-LC3 dots (**f**) were counted under florescence microscope. (**g** and **h**) H1299 and A549 cells were transfected with anti-p62 siRNA (50 nM) or control siRNA together with indicated concentrations of ESI treatment, and expression levels of the autophagy markers (**g**) and cell viability (**h**) were analyzed. Note that knockdown of p62 attenuated ESI-induced autophagy and survival. All data were representative of three independent experiments. Bars, S.E.M.; *P<0.05, **P<0.01, ***P<0.001

As both Nrf2 signaling and autophagy were activated by ESI stimuli as shown above, we then investigated the crosstalk between the two signaling pathways. It is well known that p62 works as an adaptor that binds ubiquitylated protein aggregates and delivers them to the autophagosomes.^37^ The protein level of p62 is commonly downregulated during the autophagy process. However, we unexpectedly found that p62 was increased during the ESI-induced autophagy according to the proteomic analysis (Figure 5c). As shown in Figures 5f and 7a, both mRNA and protein levels of p62 were significantly upregulated in the H1299 and A549 cells treated with ESI. Given that p62 can interact with Keap1 for degradation,^38^ we proposed that ESI could activate Nrf2 signaling and the expression of its downstream target p62, which competitively binds with Keap1, therefore rendering Nrf2 releases from Nrf2-Keap1 complex and translocates to nuclear. As shown in Figure 7b, we performed immunoprecipitation in the H1299 cells transfected with the plasmid expressing Flag-Keap1 and found that ESI indeed promoted the interaction between Keap1 and p62, and attenuated the binding of Keap1 to Nrf2, indicating a positive feedback loop of Nrf2-p62-Keap1 driven by ESI in lung cancer cells. Furthermore, the results showed that ectopic expression of p62 alone was sufficient to induce autophagy, as indicated by the increased expression levels of autophagy markers including Beclin1, ATG3 and LC3-II in H1299 and A549 cells (Figure 7c). Confocal fluorescence microscopy analysis demonstrated that overexpression of p62 enhanced punctate mCherry-LC3 staining, as compared with control group (Figures 7d–f). In addition, p62 colocalized with and enhanced the aggregation of ubiquitinated proteins (Supplementary Figure S2A), and this was further confirmed by immunoprecipitation assay, which showed that p62 overexpression led to the binding of more ubiquitinated proteins to p62 for autophagic degradation. In addition, the global ubiquitinated proteins were decreased in the p62-overexpressing cells, as compared with control group (Supplementary Figure S2B). These results strongly support that the expression of p62 could accelerate the autophagy progress. To further confirm that p62 has a critical role in ESI-induced protective autophagy, H1299 and A549 cells were treated with ESI in presence or absence of the siRNA against p62, and the results showed that knockdown of p62 significantly blocked the autophagy induced by ESI (Figure 7g). Functionally, knockdown of p62 markedly enhanced anticancer effect of ESI in lung cancer cells (Figure 7h), similar to the effect of autophagy inhibition by 3-MA (Figure 4e). Collectively, these data suggested that ESI may induce protective autophagy via an Nrf2-p62-Keap1 feedback loop in lung cancer cells.

## Discussion

In this study, we focused on the cellular response against ESI before apoptosis in lung cancer cells. Here, we provided the first evidence that ESI induces protective autophagy through Nrf2-p62-keap1 feedback loop in sustaining the survival of lung cancer cells. As our results showed that autophagy inhibitor, knockdown of Nrf2 or p62 significantly enhanced the anticancer effects of ESI, the data therefore highlight the therapeutic potential of ESI in combination with related pathway inhibitors in treatment of cancer.

Autophagy is a cellular degradation process for clearing the damaged or superfluous proteins and organelles, serving as an alternative energy source during the periods of metabolic stress to maintain homeostasis and viability.^35^ Although autophagy could be a double-edged sword, protective autophagy is frequently reported, which can sustain the survival of cancer cells.^35, 39^ Meanwhile, it has been documented that Nrf2 signaling protects cells against oxidative stress, environmental toxicants and harmful chemicals via transcriptional activation of cytoprotective genes.^23^ The crosstalk between Nrf2 signaling and autophagy remains unclear. Emerging evidences suggest that when autophagy is impaired, p62 accumulates in the cytosol and tends to competitively bind with Keap1, a negative regulator of Nrf2, resulting in prolonged Nrf2 activation,^40, 41, 42^ and that dysregulation of Nrf2 signaling pathway is able to promote cell proliferation and chemoresistance in several cancers.^43, 44, 45, 46^ In this study, we observed an autophagic flux and nuclear translocation of Nrf2 in the cells treated with ESI (Figures 2,3,4 and 5). Moreover, knockdown of Nrf2 significantly attenuated the ESI-induced autophagy, whereas blockade of the autophagy with specific inhibitor 3-MA could not affect the ESI-induced Nrf2 activation (Figure 6). These solid experimental data demonstrated that Nrf2 can act as upstream regulator of autophagy, thus uncovering a novel mechanism in which autophagy and Nrf2 are concurrently activated in cancer cells.

Deciphering the molecular events by which Nrf2 regulates autophagy is crucial for understanding of the action mechanisms of ESI and development of novel therapeutic strategies. Our data from proteomic analysis, western blot and qPCR assay suggested that p62 was upregulated at both protein and mRNA levels during ESI-induced autophagy (Figures 5f and 7a). As the target gene of Nrf2 activated by ESI, p62 may be a critical linker between Nrf2 and autophagy signaling. It has been reported that p62 is an interacting partner of Keap1, and that ectopic expression of p62 can result in the inhibition of Keap1-mediated Nrf2 ubiquitination and its subsequent degradation by the proteasome.^38^ In this regard, we showed that ESI could augment the interaction between Keap1 and p62, attenuate the binding of Keap1 to Nrf2, which allows Nrf2 to release from Keap1-Nrf2 complex and translocate to nuclear for activating target genes, such as p62 and HO-1.^36^ Our results indicate a positive feedback loop of Nrf2-p62-Keap1 driven by ESI in lung cancer cells.

**Figure 8:**
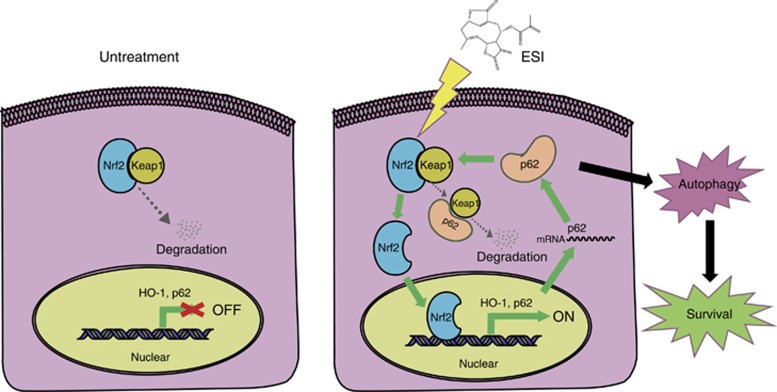
Schematic diagram summarizing the ESI-induced Nrf2-p62-Keap1 feedback loop and protective autophagy

As the product of feedback loop, accumulating p62 aggregates in cytoplasm and provokes protective autophagy in lung cancer cells. We found that overexpression of p62 enhanced the level of the membrane-bound LC3-II form ^47^ to augment autophagy (Figure 7c), and knockdown of p62 not only significantly abrogated the ESI-induced autophagy but also enhanced the anticancer effect of ESI (Figure 7h). Collectively, these data demonstrated that, in response to ESI, cellular Nrf2- p62-Keap1 feedback loop can induce protective autophagy and have an essential role in maintaining cancer cell survival (Figure 8).

Increasing evidences proved that natural products may be potential therapeutic agents for cancer treatment^48, 49, 50^ and some of the them, for example, paclitaxel, vinca alkaloids and resveratrol, have been used for chemotherapy in clinic for decades.^4, 6, 51^ ESI was previously reported to have anticancer effects by arresting G2/M phase transition, inducing ROS-dependent DNA damage and mitochondrial-mediated apoptosis,^10^ and suppressing the activity of nuclear factor-*κ*B.^52^ These evidences strongly support a promising chemotherapeutic role of ESI in cancer therapy. Interestingly, here we unexpectedly found that ESI is able to trigger an Nrf2-p62-Keap1 feedback loop, which induces protective autophagy regulatory axis for maintaining cancer cell survival. Our results showed that inhibition of autophagy or Nrf2 or p62 could markedly enhance the anticancer effect of ESI. Minimization of dosing regimens and development of combinational efficient treatment has been recognized as a trend for cancer therapy. Our study suggests a novel therapeutic strategy of combining low-dose ESI with inhibitors of Nrf2 or p62 as a potential treatment against cancers.

## Materials and methods

### Reagents and chemicals

ESI was isolated and purified in Professor Yao-Lan Li’s laboratory, College of Pharmacy, Jinan University, Guangzhou, China. Antibodies include anti-LC3A/B, anti-Beclin-1, anti-ATG3 from Cell Signaling Technology (Beverly, MA, USA), anti-p62 and anti-Keap1 from Santa Cruz (Santa Cruz, CA, USA), anti-Nrf2 and anti-ubiquitin from Abcam (Cambridge, UK). Rapmycin and 3-MA were purchased from Sigma (St.Louis, MO, USA).

### Cell lines and culture

Human lung cancer cell lines A549, H1299 and non-cancer lung epithelial cells HBE ^53^ were obtained from the American Type Culture Collection (ATCC, Manassas, VA, USA). These cells were maintained in DMEM (Life Technologies, Beijing, China) with 10% fetal bovine serum (FBS, Life Technologies) in a humidified atmosphere of 5% CO_2_ at 37 °C.

### Cell viability assay

The cytotoxic activity of ESI was measured using the WST-1 assay (Beyotime, Jiangsu, China). A549 and H1299 cells were treated with ESI at various concentrations for different time points, and then washed once and incubated with WST-1 at 37 °C for 2 h. The plates were read on an automated microplate spectrophotometer (BioTek Instruments, Winooski, VT, USA) at 450 nm.

### Colony formation assay

Cells were seeded in six-well plates at a density of 500 cells per well and cultured for 14 days under appropriate drug conditions. The plates were washed twice with PBS and fixed with methyl alcohol for 15 min at room temperature and then stained with 1% crystal violet for 5 min. All statistical measurements were acquired from three independent experiments.

### Annexin V-APC/7-ADD staining assay

Annexin V-APC/7-ADD Apoptosis Detection Kit (KeyGen, Nanjing, China) was used to determine cell apoptosis. The cells were suspended with 100 *μ*l of binding buffer (10 mM HEPES/NaOH, 140 mM NaCl, 2.5 mM CaCl_2_, pH 7.4) and stained with 5 *μ*l of APC-conjugated Annexin V and 5 *μ*l of 7-ADD for 15 min at room temperature in dark and then 400 *μ*l binding buffer was added. Apoptotic cells were analyzed by C6 flow cytometry (BD Biosciences, San Diego, CA, USA).

### Autophagy analysis

For the detection of autophasomes, H1299 cells were transfected with the plasmid expressing mCherry-EGFP-LC3 using lipofectamine 3000 (Life Technologies) according to the manufacturer’s instructions. The florescence of mCherry-LC3 or mCherry-EGFP-LC3 was detected and the fluorescence labeled vacuole formation (autophagosomes) was counted under an Olympus IX71 florescence microscope (Olympus, Tokyo, Japan). The percentage of positive cells with mCherry-LC3 or mCherry-EGFP-LC3 punctate dots was determined from three independent experiments. The cells with more than five mCherry-LC3 punctate dots were counted and a total of 30 cells were counted per treatment. Autophagic flux measuring was performed on laser scanning confocal microscopes (LSM700, Zeiss, Jena, Germany). Images were captured at × 60 magnification. DAPI staining was used to determine the morphology of cell nucleus.

### siRNA transfection

For siRNA (GenePharma Corporation, Shanghai, China) interference, cells were grown to 50% confluence in DMEM growth medium and then transfected using Lipofectamine 3000 according to the manufacturer’s instructions. Two different target siRNA sequences for each gene were listed below: 5′-CUUGCAUUAAUUCGGGAUATT-3′ and 5′- GAUGCCCAAUGUGAGAACATT-3′ for Nrf2, 5′-GUGACGAGGAAUUGACAAUTT-3′ and 5′- GGAGUCGGAUAACUGUUCATT-3′ for p62.^54^ Cells were used for follow-up experiments 24 h post-transfection.

### Subcellular fractionation

For the isolation of cytosolic and nuclear-enriched fractions, A549 and H1299 cells at 70–80% confluence were collected and washed with cold PBS twice before being resuspended in 500 *μ*l extraction buffer (10 mM HEPES-KCl (pH 7.6), 10 mM KCl, 5 mM MgCl_2_) and incubated for 10 min on ice. Next, 500 *μ*l extraction buffer with 1% Triton-100 was added to the cell supernatant to solubilize plasma membrane and leave the nuclear membrane intact. To obtain the nuclear pellet, the cell supernatant was incubated on ice for 20 min, and 500 *μ*l nuclear isolation buffer (10 mM HEPES-KCl (pH 7.6), 10 mM KCl, 5 mM MgCl_2_) was added, and then the homogenates were centrifuged at 600 *g* for 10 min at 4 °C. The supernatant fraction is the cytosolic fraction, and the pellet fraction is the enriched nuclear fraction.

### Western blot and immunoprecipitation

Proteins were extracted by RIPA lysis buffer (Cell Signaling Technology) according to the manufacturer's instructions, and the protein concentration was determined with a BCA kit (Thermo Fisher Scientific, Shanghai, China). The samples were loaded onto a 10% or 12% SDS-PAGE and subsequently electrotransferred to a PVDF membrane (Millipore, Bedford, MA, USA). The membrane was blocked with 5% nonfat milk for 1 h. After blocking, the membrane was incubated with antibody for 1 h at room temperature. After washing, the membrane was incubated with the HRP-conjugated goat anti-mouse/rabbit secondary antibodies (1 : 4000; Proteintech, Chicago, IL, USA) at room temperature for 1 h. The reaction was visualized using ECL (Bio-Rad, Hercules, CA, USA) and detected by exposure to autoradiographic film. For immunoprecipitation assay, the detailed experimental procedures were described previously.^55^

### qRT-PCR

Total RNAs were extracted using TRIzol reagent (Life Technologies). cDNA synthesis was conducted with TransScript One-Step gDNA Removal and cDNA Synthesis SuperMix (Transgen, Beijing, China) and the subsequent quantitative RT-PCR was performed on a Bio-Rad Mini Opticon real-time PCR system using iTaqTM universal SYBR Green Supermix (Bio-Rad) according to the manufacturer's instructions. Actin was included as internal control. The detailed experimental procedures were described previously.^56^ The sequences of primers were: p62, forward: 5′-AGCGTCAGGAAGGTGCCATT-3′, reverse: 5′-TTCTCAAGCCCCATGTTGCAC-3′ HO-1, forward: 5′-CATGACACCAAGGACCAGAG-3′, reverse: 5′-AGTGTAAGGACCCATCGGAG-3′.

### SILAC labeling, protein digestion and MS analyses

A549 cells were cultured for at least seven cell doublings in SILAC DMEM media (Thermo Fisher Scientific), supplemented with 10% dialytic FBS (Life Technologies) and either heavy (Lys8, Arg10) (Cambridge Isotope Laboratories, Andover, MA, USA) or light (l-lysine and l-arginine) isotope labeled amino acids. The 'light' labeled A549 cells were treated with 3.2 *μ*M ESI for 24 h, and the 'heavy' labeled A549 cells were treated with DMSO. The cells were then harvested and suspended with RIPA lysis buffer. A total of 400 *μ*g 'heavy' and 400 *μ*g 'light' protein were mixed together, filter-aided sample preparation^57^ was used for the in-solution protein digestion and high-pH RP-LC separation was performed for the peptide fractionation with minor modifications.^58^ Briefly, the mixtures were subjected to reduction and alkylation and then loaded into the ultracentrifugal filters (30 kDa cutoff; Millipore or Sartorius, Sartorius Stedim Biotech, Shanghai, China). Next, all buffer exchanges and liquid elution were carried out by centrifugation at 12 000 *g* for 15 min at 4 °C. Protein extractions were buffer exchanged with two rounds of 8 M urea and three rounds of 50 mM NH_4_HCO_3_. Then the retentate was digested using trypsin at 37 °C for 8 h. Deionized water was used to elute the peptide-rich solution, and the peptide-rich eluate was concentrated by a Speed-Vac centrifuge and resuspended with 2% acetonitrile (ACN, Sigma) in 20 mM ammonium formate before being fractionated by high-pH RP-LC. A total of 40 fractions were collected, which were then concatenated to 10 fractions, and vacuum dried before MS analyses. The peptide digests from each fraction were reconstituted in solvent A (0.1% formic acid and 2% ACN) and then analyzed using a triple-TOF 5600 MS (AB SCIEX, Framingham, CA, USA) as previously described.^58^

### Database search and bioinformatics analyses

The wiff MS data files were searched against Uniprot-Swiss Human database ((www.uniprot.org) 2016_01 Release, 20193 entries) using MaxQuant (version 1.5.2.8)^59^ with Andromeda. The parameters included: enzyme, trypsin; fixed modification, carbamidomethyl (C); variable modifications, oxidation (M), Gln→pyro-Glu (N-terminus), and acetyl (N-terminus); Precursor ion mass tolerance, 20 p.p.m.; fragment ion mass tolerance, 0.5 Da; fragment ion mass tolerance, 0.05 Da. Protein and peptide FDRs were set to 1%, and the normalized ratio of heavy *versus* light SILAC was automatically calculated by MaxQuant program. Differentially expressed proteins (DEPs) were analyzed by IPA as described previously with minor modifications.^53, 60^ Briefly, DEPs were uploaded to IPA and core analyses were performed to identify top canonical pathways. The generated networks were further optimized using ClueGO v2.2.2+CluePedia v1.1.7 programs.^61, 62^

### Statistical analysis

All assays were performed in triplicate on three independent experiments. Statistically significant differences were calculated using a Student’s *t*-test method that was performed using GraphPad Prism software (version 5.00, San Diego, CA, USA). All values were presented as the means±S.E.M. from three independent experiments. *P*<0.05 was considered statistically significant.
