# Supplementary material for: Isodeoxyelephantopin induces protective autophagy in lung cancer cells via Nrf2-p62-keap1 feedback loop
Source: Cell Death Dis. 2017 Jun 15;8(6):e2876–. doi: 10.1038/cddis.2017.265 (PMC5584574; doi:10.1038/cddis.2017.265)
Supplement: Supplementary Information [file cddis2017265x2.doc]

**Supplementary Figure S1. Effect of ESI on cell viability and growth of the non-cancer lung epithelial cells HBE.** (**A)** HBE Cells were incubated with various concentrations (up to 51.2 µM) of ESI for 24 and 48 hours and their viability were then determined by WST-1 assay. (**B**) HBE cells treated with different concentrations of ESI were compared for their abilities to form colonies. Bars, SEM; **P* < 0.05, ***P* < 0.01, ****P* < 0.001.

**Supplementary Figure S2. Ubiquitinated proteins bind to p62 for autophagic degradation.**

**(A, B)** H1299 cells were transfected with pEGFP-p62 plasmid or pEGFP-N1 (vector control) for 24 hours. The ubiquitinated proteins were stained with red fluorescence (A); Bar = 10 μm. Immunoprecipitation was performed using an anti-p62 antibody, and immunublotting was carried out on the total cell lysates or immunoprecipitates with the indicated antibodies including ubiquitin and p62 (B). Actin and IgG (H) were included as loading control for quantitation. All data were representative of three independent experiments. Bars, SEM; **P* < 0.05.
