# Supplementary figures and images for: Isodeoxyelephantopin induces protective autophagy in lung cancer cells via Nrf2-p62-keap1 feedback loop
Source: Cell Death Dis. 2017 Jun 15;8(6):e2876–. doi: 10.1038/cddis.2017.265 (PMC5584574; doi:10.1038/cddis.2017.265)

A

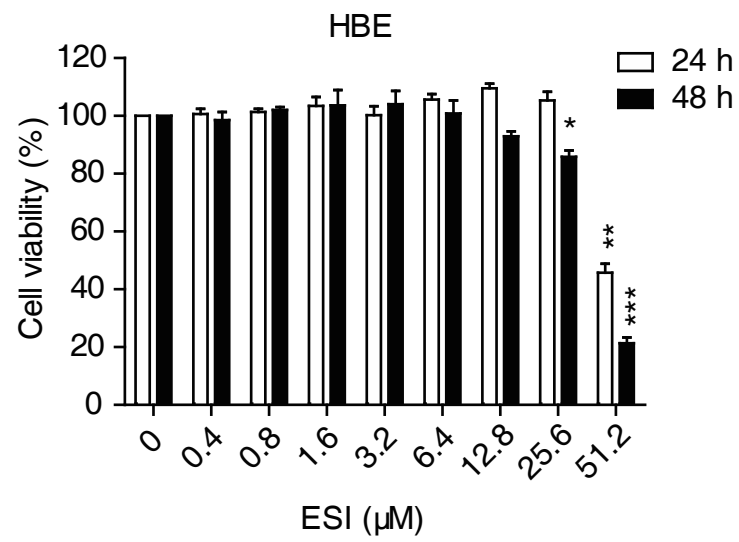

B

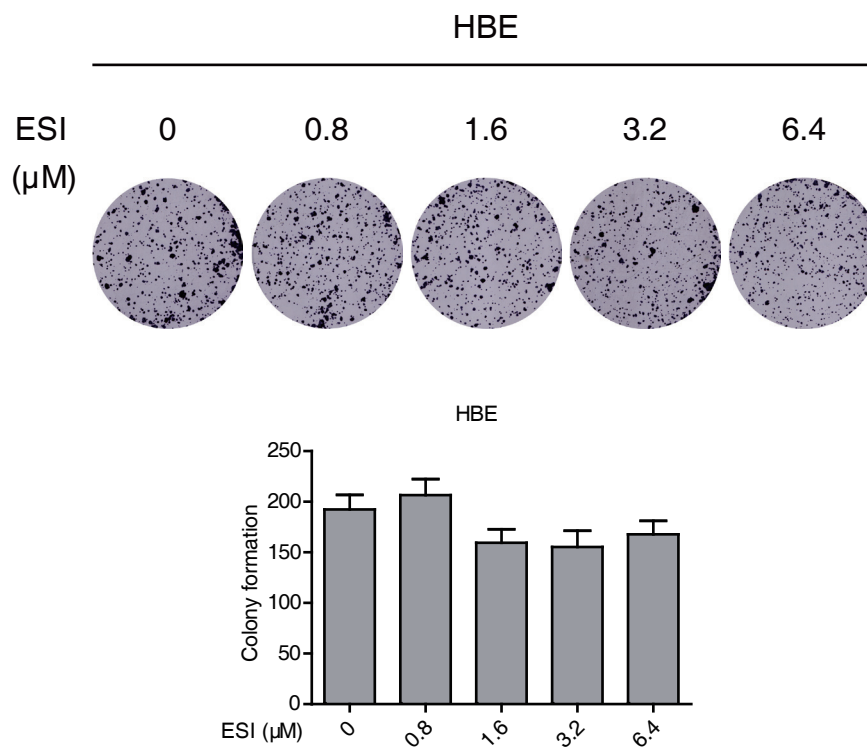

Supplementary Figure S1

Supplement: Supplementary Figure 1 [file cddis2017265x3.pdf]

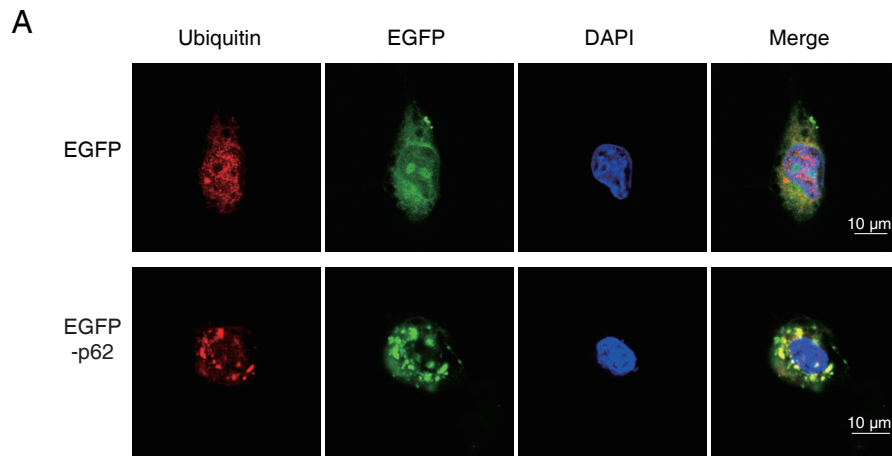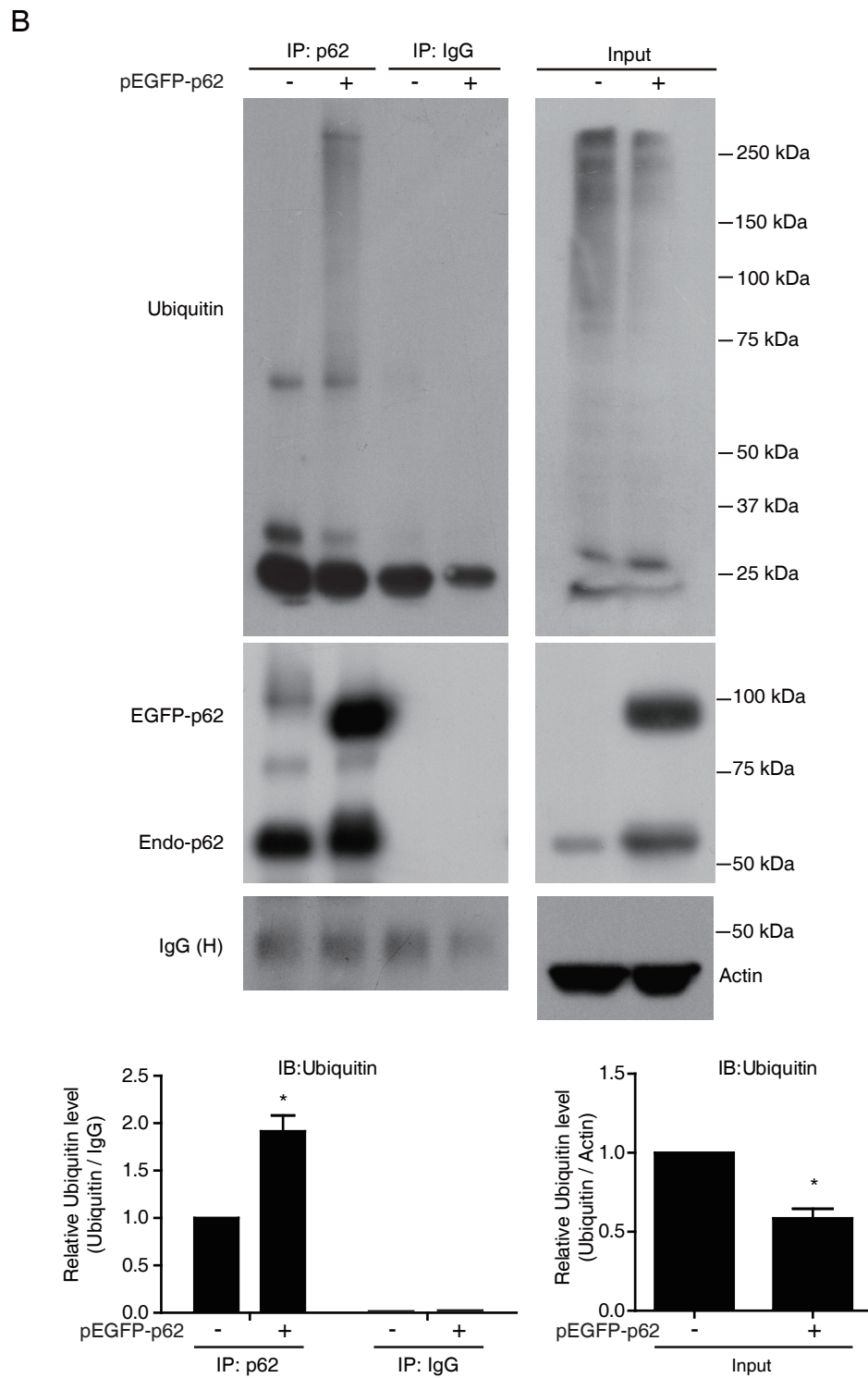

Supplementary Figure S2

Supplement: Supplementary Figure 2 [file cddis2017265x4.pdf]
